# Hand-to-Face Remapping But No Differences in Temporal Discrimination Observed on the Intact Hand Following Unilateral Upper Limb Amputation

**DOI:** 10.3389/fneur.2017.00008

**Published:** 2017-01-20

**Authors:** Kassondra L. Collins, Danielle L. McKean, Katherine Huff, Mark Tommerdahl, Oleg Vyacheslavovich Favorov, Robert S. Waters, Jack W. Tsao

**Affiliations:** ^1^Department of Neurology, University of Tennessee Health Science Center, Memphis, TN, USA; ^2^Department of Anatomy and Neurobiology, University of Tennessee Health Science Center, Memphis, TN, USA; ^3^Department of Biomedical Engineering, University of North Carolina at Chapel Hill, Chapel Hill, NC, USA; ^4^Children’s Foundation Research Institute, Le Bonheur Children’s Hospital, Memphis, TN, USA

**Keywords:** amputation, tactile discrimination, referred sensation, phantom limb, reorganization, somatosensory

## Abstract

Unilateral major limb amputation causes changes in sensory perception. Changes may occur within not only the residual limb but also the intact limb as well as the brain. We tested the hypothesis that limb amputation may result in the detection of hand sensation during stimulation of a non-limb-related body region. We further investigated the responses of unilateral upper limb amputees and individuals with all limbs intact to temporally based sensory tactile testing of the fingertips to test the hypothesis that changes in sensory perception also have an effect on the intact limb. Upper extremity amputees were assessed for the presence of referred sensations (RSs)—experiencing feelings in the missing limb when a different body region is stimulated, to determine changes within the brain that occur due to an amputation. Eight of 19 amputees (42.1%) experienced RS in the phantom limb with manual tactile mapping on various regions of the face. There was no correlation between whether someone had phantom sensations or phantom limb pain and where RS was found. Six of the amputees had either phantom sensation or pain in addition to RS induced by facial stimulation. Results from the tactile testing showed that there were no significant differences in the accuracy of participants in the temporal order judgment tasks (*p* = 0.702), whereby participants selected the digit that was tapped first by a tracking paradigm that resulted in correct answers leading to shorter interstimulus intervals (ISIs) and incorrect answers increasing the ISI. There were also no significant differences in timing perception, i.e., the threshold accuracy of the duration discrimination task (*p* = 0.727), in which participants tracked which of the two digits received a longer stimulus. We conclude that many, but not all, unilateral upper limb amputees experience phantom hand sensation and/or pain with stimulation of the face, suggesting that there could be postamputation changes in neuronal circuitry in somatosensory cortex. However, major unilateral limb amputation does not lead to changes in temporal order judgment or timing perception tasks administered *via* the tactile modality of the intact hand in upper limb amputees.

## Introduction

Great debate ensues regarding the etiology of phantom limb sensations (PLSs) and associated pain. Almost all amputees experience PLSs (1, 2). The minority that tends not to experience any phantom sensations typically includes congenital amputees (3, 4), although one study has identified such experiences in this population (5). More than 80% of all amputees will also experience phantom limb pain (PLP), characterized by electric shock, stabbing, and cramping sensations (6). PLP is a debilitating condition for many amputees. Unfortunately, the mechanisms that create phantom experiences, including sensation and pain, are not understood. When an amputation occurs, the peripheral limb is removed from the body causing drastic changes not only in the peripheral but also in the central nervous systems. Muscles and nerves attempt to forge new connections in place of lost ones, causing reorganization within the residual limb and the brain (7). Several imaging studies have shown that, after an amputation, cortical representations of adjacent remaining body parts take over the cortical area that once responded to the now amputated region. In particular, the face-representing somatosensory cortical region expands and takes over the arm area in upper extremity amputees (3, 8–11).

Determining the mechanisms and specific pathways within the brain that are affected by an amputation will lead to further insight regarding the experience of PLS and pain. This study aimed to investigate the changes that occur within the brain of upper extremity amputees, testing the hypothesis that upper extremity amputees will experience hand-to-face remapping. Through the utilization of temporally based tactile stimulation, we also examined potential sensory perception changes that could occur within pathways controlling the intact limb. This study aimed to confirm previous studies on hand-to-face remapping while also determining other factors that may play a role in such experiences.

## Participants and Methods

### Participants

Participants for this study included 19 unilateral upper extremity amputees (Table 1) and 27 normal control participants. Control participants were recruited through the University of Tennessee Health Science Center from July 2015 through July 2016, and amputee participants were recruited at the National Amputee Coalition conferences in Tucson, AZ (July 2015) and Greensboro, NC (June 2016). The Institutional Review Board at the University of Tennessee Health Science Center gave approval for the study, and all participants provided written informed consent. Inclusion criteria, except for the presence of an amputation, were the same for all groups and included being between the ages of 18 and 65, not having brain injury, able to follow instructions, and normal or corrected-to-normal vision. Exclusion criteria included evidence or the history of a major medical, neurological, or psychiatric illness, any traumatic brain injury, a learning disability, and drug or alcohol abuse/dependence within the last 3 months, except nicotine, taking prescription drugs or supplements that might affect brain function, and having serious vision or hearing problems.

**Table 1:** **Unilateral upper extremity amputee participant information**.

Participant #	Amputation	Cause	RS	PLS	PLP
1	RAE	Trauma	Yes	Yes	Yes
2	RBE	Trauma	Yes^b^	No	No
3	RBE	Trauma	No	No	No
4	RBE	Trauma	No	Yes	No
5	LBE	Congenital	Yes	Yes	No
6	LBE	Trauma	Yes	Yes	No
7	LBE	Congenital	No	Yes	No
8	LAE	Trauma	Yes	Yes	Yes
9	LBE	Congenital	No	Yes^a^	No
10	LAE	Trauma	No	Yes	Yes
11	RBE	Trauma	Yes^b^	No	No
12	LBE	Trauma	No	Yes	Yes
13	RAE	Trauma	No	Yes	Yes
14	RBE	Trauma	No	Yes	Yes
15	RBE	Trauma	Yes	Yes	Yes
16	LBE	Trauma	No	Yes	No
17	RAE	Cancer	Yes	Yes	Yes
18	RBE	Compartment syndrome	No	Yes	Yes
19	RAE	Trauma	No	Yes	Yes

*Data include participant number, location of the amputation (R, right; L, left; AE, above elbow; BE, below elbow), cause of the amputation, whether or not they experienced referred sensation (RS), phantom limb sensation (PLS), and/or phantom limb pain (PLP)*.

*^a^Reported feeling a “fizzy” sensation in the missing limb*.

*^b^Phantom sensation only brought on by mislocalization of touch*.

### Hand-to-Face Remapping

Amputees were asked to complete a series of questions regarding their amputation and phantom experiences. Information regarding the time since the amputation, the experience of phantom sensations, and the experience of PLP were investigated. Nineteen upper extremity amputees participated in facial mapping in order to determine how the brain reorganizes sensations as a result of amputation. The facial responses experienced by each amputee were mapped by using a stimulus consisting of a Q-tip brushed over different areas of the face. Testing began over the forehead with short smooth brushes and then moved around the eye, down the cheek, and over the chin. Brushing was completed both contralateral and ipsilateral to the side of amputation. As the investigator was brushing the face, the amputee was instructed to verbally express where the location of the brushing was felt. If facial mapping caused sensations within the phantom limb, repeat testing with a Q-tip dipped in cold water was performed. Finally, participants attempted to map the sensations on their own. All verbal reports of sensation felt within the phantom limb were recorded and the location(s) identified.

### Tactile Testing

Tactile stimuli were delivered to two fingers with a custom-built tactile stimulator (Cortical Metrics, Carrboro, NC, USA). Control participants underwent a battery of testing conducted on the index and middle fingers of both right and left hands. Upper extremity amputees completed the testing with *the intact hand*. Testing included temporal order judgment and duration discrimination tasks. During the testing session, the participants were situated with their arm (right then left for controls, intact arm for amputees) on a wrist support and fingers positioned appropriately over the tactile stimulator. Mechanical stimulation was applied to the tips of the index and middle fingers. A computerized procedure guided participants through a series of questions, answered *via* verbal report and recorded by a research member, relating to what the participants perceived on the tips of each finger. In both of the tasks described below, a simple tracking paradigm was used to determine each participant’s difference limen, the amount that the stimulus must be changed in order for differences between finger perceptions to be detected. Visual cues on the computer screen informed participants about appropriate times to provide their response. Practice trials were performed before each test to allow the participants to become familiar with the test, and three consecutive correct responses to the training trials were required before data acquisition began. The participant was not provided with feedback or knowledge or response accuracy during data collection trials.

### Temporal Order Judgment

To assess temporal order judgment, two taps were delivered sequentially, one to each finger, with an initial interstimulus interval (ISI) of 150 ms. Participants were queried as to which of the two stimuli came first. Subsequently, as the result of the subjects’ response, the ISI was altered between each trial. The tracking paradigm employed resulted in correct answers leading to shorter ISIs and incorrect answers increasing the ISI. For each trial, the finger that received the first of the two pulses was chosen randomly. Subjects were required to report which finger was tapped first.

### Duration Discrimination

Duration discrimination is the minimal difference in durations of two stimuli at which an individual can successfully identify the stimulus that has a longer duration. Sequential stimulus vibrations of varying durations were delivered, one to each finger. Subjects were asked to report which of the two fingers received the longer stimulus duration. The “standard” stimulus lasted 500 ms and at the start the “test” stimulus lasted 750 ms. Discrimination threshold determination was assessed using the same tracking paradigm, which reduced the duration of the test stimulus when subjects answered correctly and increased the duration of the test stimulus when the responses were incorrect. The finger and order of the stimuli were chosen at random for each trial.

### Data Analysis

ANOVA was used to analyze results of tactile testing comparing between upper extremity subject groups and controls. Analyses conducted on the experience of referred sensation (RS) and the presence of PLP were also completed utilizing direct participant verbal reports, a 2 × 2 factorial ANOVA test and a Pearson’s correlation test. Significance was determined by a *p* value <0.05.

## Results

### Hand-to-Face Remapping

**Figure 1:**
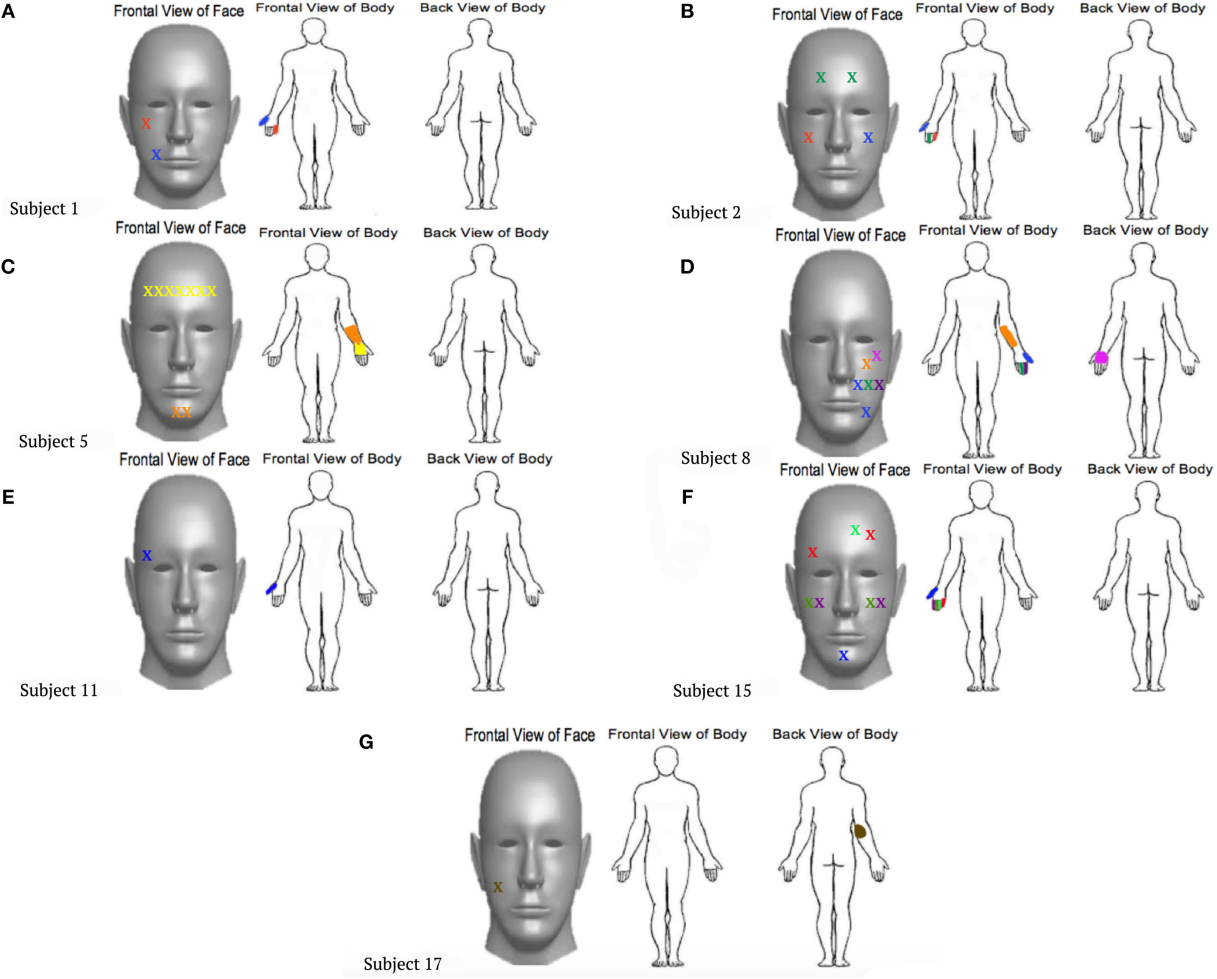
**Locations touched on the faces that were reported to also elicit sensations within the phantom limb**. Each point marked by an “X” on the face corresponds to a location that was felt on the phantom limb, represented in the same color. **(A)**–**(G)** represent the individual experiences felt by different participants, labeled by subject numbers that correspond to Table 1.

Eight out of 19 (42.1%) upper extremity amputees, including one congenital participant, experienced a mislocalization of touch when an area of their face was brushed. Similar to the results reported by Ramachandran and Rogers-Ramachandran (10), points on the face of each participant who reported elicited sensations within the phantom limb were documented and marked on a forelimb and face diagram, indicating the appropriate body region (Figure 1). The cheek area evoked the greatest number of RSs. Two participants experienced the feeling of their little finger when the cheek was brushed. Two participants also reported feeling the first finger when the cheek was stimulated. In addition, amputees reported feeling the thumb, back of hand, underside of the arm, and elbow through cheek stimulation. Three participants reported mislocalization of touch when the forehead was stimulated, expressing feelings within the third finger, palm, and thumb. When the chin was brushed, two participants experienced RSs of the thumb and palm. Four of the participants only experienced phantom sensations in the amputated limb when the ipsilateral side of the face to the amputation was stimulated. Two participants experienced sensations in the amputated limb when either side of the face was stimulated, and one felt sensations when more of the center of the face was stimulated.

After the identification of prominent mislocalization of touch, investigators attempted to remap the experiences with a Q-tip dipped in cold water. Only one participant felt that the sensations were more intense with the cold water, all other participants reported the same experiences as felt with the dry Q-tip. Once the cold-water test was completed, the subjects were then asked to conduct the facial mapping on themselves. Two participants reported being able to still feel RSs, while six participants no longer felt any RSs.

In addition to obtaining reports of the mislocalization of touch, investigators also asked participants to report the time since amputation and their experiences with phantom sensation or PLP. Statistical analysis conducted on the time since amputation and the presence of RS showed no correlation, with the average time since amputation being 211.26 months (*p* = 0.507). A 2 × 2 factorial ANOVA determined that there was no significant correlation between any of the experiences and the presence of RS (*p* = 0.134). A Pearson’s correlation test confirmed these results as well (*p* = 0.134). Out of the eight upper extremity amputees who experienced RS, four had PLP and four reported no PLP. In addition, six regularly experienced phantom sensations prior to testing and only two did not. Also, 6 of the 11 amputees who did not experience RSs also experienced PLP, and 10 experienced PLS, with only 1 not experiencing the presence of the missing limb.

Amputees who do not experience PLSs tend to be congenital amputees (those born without a limb) (3, 4). Three congenital amputees completed the hand-to-face remapping study and reported their experiences with phantom sensations and PLP. Initially, all three congenital upper extremity amputees self-reported no RSs, no phantom sensations, and no PLP. However, two of the three participants, when asked to graphically depict their phantom limb on a piece of a paper, completed the task, suggesting that they do, in fact, feel the presence of a phantom limb. The one congenital amputee who was unable to trace the phantom limb reported that they did not have phantom sensation, just a “fizzy” feeling, again implying the feeling of sensation within the missing limb. Additionally, while conducting the hand-to-face remapping task, one of the congenital amputees who was able to depict their phantom limb felt the brushing sensation within the palm of the phantom limb when the forehead and chin were stimulated. This information is important to note, considering the rarity of phantom sensation reported by congenital amputees.

### Temporal Order Judgment

In the temporal order judgment task, in which the participant was instructed to determine which digit experienced a test tap stimulus first, the mean threshold scores were 31.4 ± 19.5 and 34 ± 22.8 ms for the control and upper extremity amputees, respectively (*p* = 0.702). Additional analysis was conducted to determine correlations between threshold scores and whether the left or right arm was amputated. When compared to controls, neither right nor left-arm amputees differed in the temporal order judgment task (*p* = 0.668).

### Duration Discrimination

The threshold accuracy of the duration discrimination task was determined to investigate the potential changes in the accuracy of timing perception for upper extremity amputees. In the duration discrimination task, participants were asked to identify which digit received a longer stimulus. The mean threshold scores were 66 ± 25.5 and 69.1 ± 28.3 ms for control and upper extremity amputees, respectively (*p* = 0.727). When the amputees were separated based on the side of their amputation, results showed no significant difference in the scores obtained on the duration discrimination task (*p* = 0.204).

## Discussion

When an individual loses a limb, many changes occur, not only within the peripheral system but also within the central nervous system. Although descriptions of phantom sensations and phantom pain have been around since at least the sixteenth century (12), the etiology of these experiences is still not understood. After an amputation occurs, the nerves and muscles attempt to build connections wherever possible, leading to reorganization within the residual limb. Whether this reorganization fuels the central nervous system reorganization or *vice versa* needs further investigation. Results from this study indicate that cortical reorganization may be confined to the contralateral somatosensory cortex and does not significantly affect other cortical areas or spread transcallosally to the somatosensory cortex in the opposite hemisphere.

Our investigation of the effects of an amputation on the cortex were conducted through the use of facial mapping. By using a Q-tip to brush areas of an amputee’s face and evoking phantom sensations in the missing limb, we were able to positively identify hand-to-face remapping in 42.1% of upper extremity amputees. Such results show that the removal of an upper extremity does indeed cause changes within the main cortical target of somatosensory input projections, the somatosensory cortex. Additionally, this study showed that cortical reorganization is not always directly linked to the experience of PLP, since half of those experiencing mislocalization of touch failed to report any PLP. The time since amputation also did not play a role in the experience of cortical reorganization. Results are encouraging, if not definitive, and provide an important first step for future studies involving the timing of the onset and overall plasticity of cortical reorganization. One very interesting finding arising from this study was the identification of a congenital amputee who experienced mislocalization of touch. Facial mapping caused sensation within the palm of the missing limb as the forehead and chin were brushed. Initially, this participant reported that they did not experience PLP or a phantom limb; however, when asked to depict the phantom limb on a piece of paper, they proceeded to trace around a limb that they still perceived, a phantom representation. The ability of this individual to feel the palm of the missing limb on their forehead and chin shows that the brain has undergone cortical reorganization. These findings raise questions about the cause of the congenital limb loss. Although this person was born without the limb, there is the possibility that the limb was formed *in utero* and then removed, such as from amniotic band syndrome. In this scenario, there was regression of the limb during development such that the limb representation developed within the brain and did not disappear when the limb was lost. If the limb never formed during development, it is possible that the cortex still maintains some innate representation of all body parts. Such findings go against multiple studies that indicate congenital amputees do not experience phantom limbs and/or RS due to cortical reorganization (3, 4, 8). Furthermore, since there was no correlation between the presence of phantom pain and whether there was detectable hand-to-face remapping, these findings suggest that cortical reorganization alone is not the etiology of phantom pain as previously postulated (13–15).

Furthermore, temporally based tactile stimulation testing was completed on upper extremity amputees to determine the effects of amputation on the temporal processing in the CNS. Temporal order judgment task and duration discrimination task are timing tests that are controlled by areas of the brain other than the somatosensory cortex. As described by multiple studies, the ability to judge which finger receives the first test pulse is controlled mainly by the pre-supplementary motor area and posterior parietal cortex (16–18). Duration discrimination, the ability to determine which test pulse lasted longer, is thought to reflect activity predominantly centered in the cerebellum (19). Results from tactile testing on the intact limb of upper extremity amputees and controls showed that there is no significant difference on timing perception tasks between the two groups. These findings suggest that amputations lead to remapping effects that do not have an impact on timing measures that take place outside of the denervated somatosensory cortex or changes within pathways controlling the intact limb.

For clinical purposes and the management of PLP, more effort into determining the utility of visualization and residual limb movement therapies is necessary, especially if cortical reorganization alone is not a key factor in the presence of phantom pain. Future research efforts should focus on the timing of cortical reorganization to gain more insight into whether the peripheral or central nervous systems cause and/or maintain the phantom experiences. Additionally, tactile testing targeting the somatosensory cortex contralateral to the amputated side will provide information regarding changes there and effects that therapies and treatments contribute to these changes. Determining the effects that an amputation has on the organization of the brain will enable researchers to gain further knowledge about the presence of PLSs. Finally, more research needs to be conducted on the experience of phantom sensations felt by congenital amputees. It is possible that determining the factor that causes a congenital amputee to experience phantom sensations may lend great insight to the understanding of overall phantom experiences.

## Ethics Statement

This study was carried out in accordance with the recommendations of the human research protection guidelines, University of Tennessee Health Science Center IRB, with written informed consent from all subjects. All subjects gave written informed consent in accordance with the Declaration of Helsinki. The protocol was approved by the University of Tennessee Health Science Center IRB.

## Author Contributions

KC assisted with running of participants, assisted with data analysis, and lead manuscript creation. DM oversaw and completed the running of participants and completed data analysis. KH assisted with running of participants and analyzing data. MT and OF designed the protocol, completed data analysis, and assisted with the manuscript. RW assisted with data analysis, manuscript creation, and editing of the manuscript. JT oversaw study design and execution and led manuscript editing.

## Conflict of Interest Statement

MT is cofounder of Cortical Metrics, the company that built the tactile stimulator used in the study. No other authors have a conflict of interest.
